# O-GlcNAcylation dictates pyroptosis

**DOI:** 10.3389/fimmu.2024.1513542

**Published:** 2024-12-17

**Authors:** Yue Lang, Jincheng Li, Leiliang Zhang

**Affiliations:** ^1^ Department of Clinical Laboratory Medicine, The First Affiliated Hospital of Shandong First Medical University and Shandong Provincial Qianfoshan Hospital, Jinan, Shandong, China; ^2^ Department of Pathogen Biology, School of Clinical and Basic Medical Sciences, Shandong First Medical University and Shandong Academy of Medical Sciences, Jinan, Shandong, China

**Keywords:** pyroptosis, O-GlcNAcylation, GSDMD, GSDME, NLRP3

## Abstract

O-GlcNAcylation is a dynamic post-translational modification involving the attachment of N-acetylglucosamine to serine and threonine residues. This review emphasizes its role in regulating the signaling pathways of pyroptosis. Specifically, the O-GlcNAcylation of GSDMD is linked to the modulation of pyroptosis, suggesting that enhancing O-GlcNAcylation of GSDMD could be crucial for improving hypoperfusion in sepsis. Additionally, GSDME, another member of the gasdermin family, facilitates macrophage pyroptosis through O-GlcNAcylation induced by high glucose levels in the context of periodontitis. The review also examines the effects of O-GlcNAcylation on the NLRP3 inflammasome and its regulators, including NEK7 and NF-κB. Overall, this review emphasizes the role of O-GlcNAcylation in the pathogenesis of conditions such as sepsis, periodontitis, and osteoarthritis, identifying potential therapeutic targets for managing inflammatory responses through its targeted modulation.

## 1 Introduction

**Figure 1:**
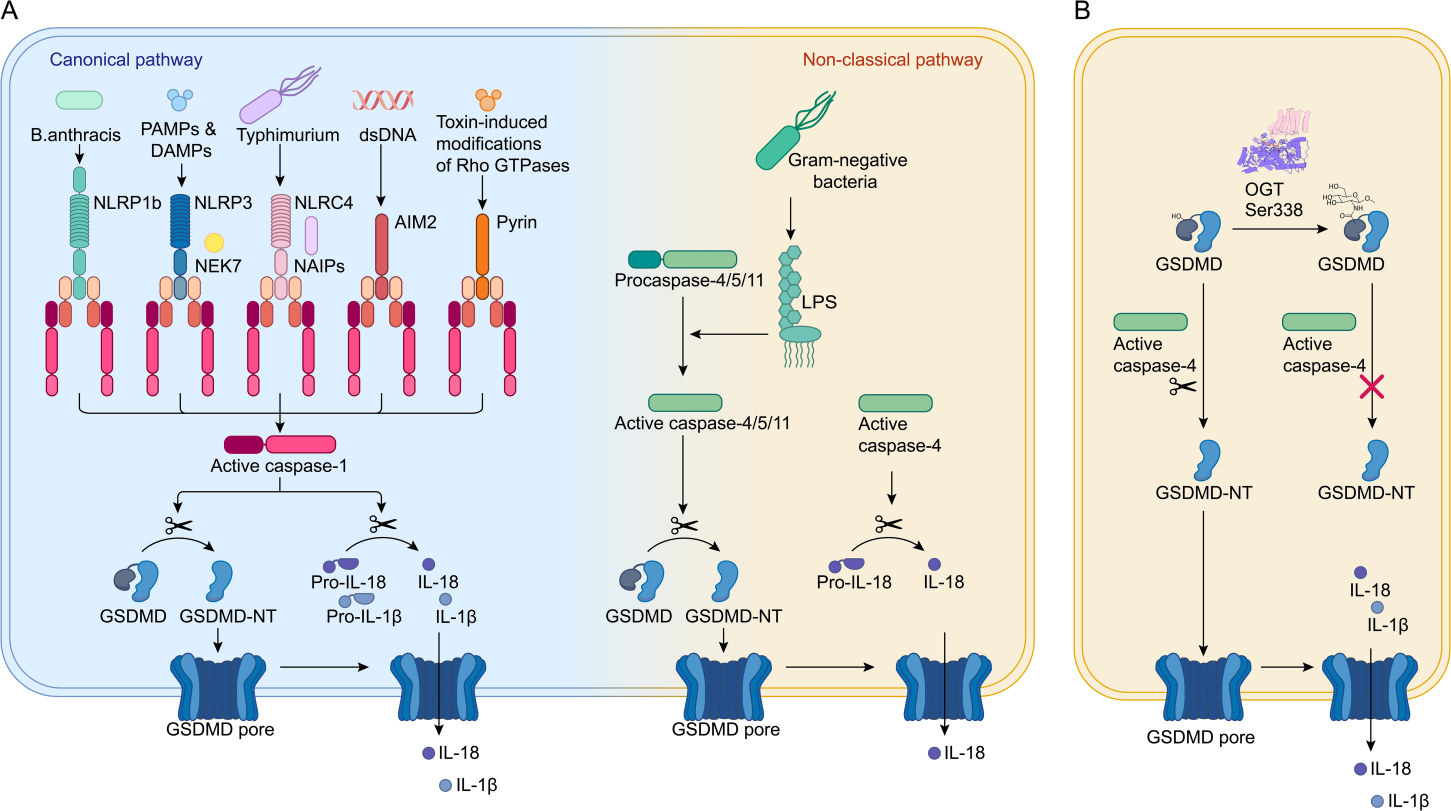
O-GlcNAcylation of GSDMD. **(A)** In the canonical pathway, pathogen-associated signals are detected by PRRs like NLRP1, NLRP3, NAIPs, AIM2, and Pyrin. These PRRs then activate pro-caspase-1 through ASC or NLRC4 adaptors, resulting in the formation of active caspase-1, which cleaves GSDMD and pro-IL-1β/18 into IL-1β/IL-18 and GSDMD-N. GSDMD-N moves to the plasma membrane, forms pores, causing cell swelling and lysis, and facilitates the release of mature IL-1β/IL-18, leading to cell pyroptosis. In the non-canonical pathway, pro-caspase-4/5/11 are directly activated by binding to LPS, leading to cleavage of GSDMD into GSDMD-N and the initiation of pyroptosis. **(B)** GSDMD is modified by O-GlcNAc at the Ser338 site. This modification prevents the interaction between caspase-4 and GSDMD, reducing the levels of GSDMD-N and thereby inhibiting cell pyroptosis.

Pyroptosis is a form of regulated cell death that fundamentally relies on the formation of plasma membrane pores by members of the gasdermin (GSDM) protein family, which is typically (though not exclusively) triggered by the activation of inflammatory caspases (1). This process involves GSDM-N oligomerization and pore formation, leading to cell lysis and the subsequent release of inflammatory cytokines IL-1β and IL-18 (**Figure 1A**). Pyroptosis is highly lytic and pro-inflammatory, distinguishing it from other forms of cell death. In humans, the GSDM family consists of six paralogs: GSDMA, GSDMB, GSDMC, GSDMD, GSDME (also known as DFNA5), and PJVK (also known as DFNB59) (2, 3). Except for DFNB59, all these paralogs are involved in regulating pyroptosis (2, 3). The primary mechanism through which GSDMs drive pyroptosis is via their N-terminal pyroptosis-inducing domain, which facilitates the formation of pores in the cell membrane, leading to cell death and the release of pro-inflammatory cytokines. This activity underscores the critical role of GSDMs in the innate immune response and their potential implications in various inflammatory diseases (2).

The nucleotide-binding domain and leucine-rich repeat-containing protein 3 (NLRP3) inflammasome is a critical component of the host’s intracellular defense system, serving as a key mechanism to prevent pathogen invasion (4). It comprises three primary components: NLRP3, caspase-1, and apoptosis-associated speck-like protein containing a caspase recruitment domain (ASC). The activation of the NLRP3 inflammasome triggers the proteolytic cleavage of procaspase-1 into its active form, caspase-1, which then facilitates the secretion of pro-inflammatory cytokines IL-1β and IL-18 (**Figure 1A**) (5). These cytokines play a central role in initiating the innate immune response and inflammation. Additionally, mammalian NIMA-related kinases (NEKs) are a family of serine/threonine kinases, consisting of NEK1 through NEK11. NEK7, the smallest member of this family, comprises only a catalytic domain and a 30–40 amino acid N-terminal extension. NEK7 has been shown to be crucial for the activation of the NLRP3 inflammasome through direct interaction, highlighting its significant role in the regulation of inflammation and immune responses (6, 7). The NF-κB family of transcription factors, which includes p65 (RelA), RelB, c-Rel, p105 (NF-κB1), and p100 (NF-κB2), plays a crucial role in regulating the functions of various immune cells (29). NF-κB acts as a pivotal mediator in initiating the priming signal required for NLRP3 inflammasome activation, orchestrating the transcriptional upregulation of NLRP3 and pro-IL-1β in response to various pattern recognition receptors (PRRs) ligands and cytokines (33, 34).

O-linked-β-N-acetylglucosamine (O-GlcNAc) glycosylation (O-GlcNAcylation) is the reversible addition of a single O-GlcNAc monosaccharide to various cytoplasmic, nuclear, and mitochondrial proteins (8). Glycosylation has traditionally been regarded as a stable and conserved post-translational modification. O-GlcNAcylation represents a specialized form of glycosylation characterized by its reversible and transient nature, making it more analogous to phosphorylation. However, unlike phosphorylation, which is regulated by a diverse array of kinases and phosphatases, O-GlcNAcylation is controlled by a single pair of enzymes: O-GlcNAc transferase (OGT) and O-GlcNAc-selective N-acetyl-β-d-glucosaminidase (OGA) (8, 9). This modification is facilitated by OGT, which transfers N-acetyl-glucosamine from UDP-GlcNAc to serines or threonines on target proteins (10–12). The reversal of O-GlcNAcylation is carried out by OGA, which selectively removes O-GlcNAc from modified proteins (10).

O-GlcNAcylation affects various biochemical properties of protein substrates, including protein phosphorylation and stability (13, 14). This modification occurs on serine (S) and threonine (T) residues, which overlap with sites of protein phosphorylation, suggesting a potential competition or cooperation between O-GlcNAcylation and phosphorylation. O-GlcNAcylation is implicated in various immune cells, including T cells, B cells, natural killer (NK) cells, macrophages, and neutrophils (15). It promotes neutrophil mobilization by increasing Rac activation, negatively regulates the cytotoxic effects of NK cells, and modulates the activation of T cells and mature B cells (15). Regarding the inflammatory response, O-GlcNAcylation exhibits dual effects—both promoting and inhibiting inflammation. The determining factor for whether a specific O-GlcNAcylation modification has a pro- or anti-inflammatory role depends on the type of insult present (16). For instance, in the context of high-glucose (HG) concentrations associated with diabetes, O-GlcNAcylation plays a pro-inflammatory role in the NF-κB signaling pathway. In relation to cardiac and vascular diseases, O-GlcNAc modification of proteins in the vasculature may represent a novel anti-inflammatory and vasoprotective mechanism. Furthermore, O-GlcNAcylation modulates inflammation by sensing the energy states related to normal and excess nutrient levels (16).

Recent research has highlighted the growing importance of O-GlcNAcylation in the regulation of pyroptosis-related proteins, including NLRP3, GSDMD, GSDME, and NEK7. Exploring the interplay between pyroptosis and O-GlcNAcylation could help elucidate the intricate mechanisms governing innate immunity and disease pathogenesis. This review aims to examine the current understanding of how O-GlcNAcylation affects these proteins within the pyroptosis pathway, uncovering potential mechanisms and therapeutic opportunities for managing immune responses and treating inflammatory diseases. By delving into the complex relationship between cell death and O-GlcNAcylation, new therapeutic targets may emerge in the field of immunology.

## 2 O-GlcNAcation in pyroptosis pathway

### 2.1 GSDMD

GSDMD serves as a key executor of pyroptosis (17). It can be activated through two distinct pathways. The canonical pathway involves the recognition of pathogens or damage by PRRs, including nucleotide-binding domain and leucine-rich repeat-containing protein 1 (NLRP1), NLRP3, NAIPs (NLR family apoptosis inhibitory proteins), absent in melanoma 2 (AIM2), and Pyrin (**Figure 1A**). These PRRs then activate pro-caspase-1 through ASC or NLRC4 adaptors, resulting in the formation of active caspase-1, which cleaves pro-IL-1β/18 and GSDMD into IL-1β/IL-18 and the N-terminal domain of GSDMD (GSDMD-N). The non-canonical pathway, on the other hand, relies on caspase-11 in mice and caspase-4/5 in humans, which are directly activated by lipopolysaccharides (LPS) (**Figure 1A**) (18, 19). GSDMD-N translocates to the plasma membrane, causing membrane perforation and ultimately leading to cell pyroptosis, accompanied by the release of inflammatory cytokines such as IL-1β and IL-18 (19).

Recent research has shed light on how O-GlcNAc modification of GSDMD can influence pyroptosis in the in the pathogenesis and progression of sepsis (17). This study discovered that endothelial cell pyroptosis is crucial in sepsis-related endothelial injury and hypoperfusion. GSDMD, which acts as an executor of pyroptosis, undergoes O-GlcNAcylation, a modification that seems to reduce the occurrence of cell pyroptosis. In human umbilical vein endothelial cells (HUVECs) treated with LPS, a decrease in O-GlcNAc levels was observed in conjunction with an increase in LPS concentration. This was associated with pronounced morphological changes and increased lactate dehydrogenase release, indicative of pyroptosis. Notably, these effects were attenuated by thiamet G (TMG), suggesting that TMG may inhibit cell pyroptosis by preventing GSDMD cleavage. TMG treatment resulted in reduced levels of GSDMD-N, implying that TMG may inhibit pyroptosis by interfering with GSDMD processing (17). Further investigation revealed that O-GlcNAcylation occurs on GSDMD, leading to a reduction in GSDMD-N levels. This was supported by TMG treatment, which demonstrated that O-GlcNAcylation of GSDMD inhibited its cleavage, leading to a reduction in the levels of the pyroptosis effector molecule GSDMD-N (**Figure 1B**) (17). Interestingly, TMG also inhibited the interaction between GSDMD and caspase-4, suggesting that O-GlcNAcylation affects GSDMD cleavage by blocking the interaction between GSDMD and caspase-4 (17).

To identify the specific O-GlcNAcylation sites on GSDMD, multiple prediction tools were employed. Ser-338 was identified as a potential site for O-GlcNAc modification (**Figure 1B**) (17). Mutations at Ser-338 affected GSDMD O-GlcNAcylation, confirming it as a modification site. Subsequent experiments using the S338A mutant plasmid in co-immunoprecipitation (Co-IP) assays demonstrated that TMG treatment enhanced GSDMD O-GlcNAcylation, which reduced the binding between GSDMD and caspase-4 as well as GSDMD cleavage (17).

In summary, O-GlcNAc modification alleviates cell pyroptosis by disrupting the interaction between GSDMD and caspase-4, leading to decreased levels of GSDMD-N. This finding provides evidence that O-GlcNAcylation, as a novel post-translational modification of GSDMD, is beneficial for maintaining organ blood flow perfusion during sepsis. Diminished blood flow perfusion is one of the factors contributing to increased mortality in septic patients. Enhancing the O-GlcNAcylation of GSDMD presents potential therapeutic strategies for modulating inflammatory diseases. Prior studies have shown that mice with a conditional knockout of OGT in macrophages, exhibit significantly increased RIPK3 activation, elevated synthesis of pro-inflammatory cytokines, and greater susceptibility to mortality in experimental sepsis compared to control mice (20). This suggests that the pathogenesis of sepsis is closely linked to the role of O-GlcNAcylation.

### 2.2 GSDME

GSDME is a member of the GSDM family, and its activation promotes pyroptosis in various diseases by increasing inflammatory levels. Periodontitis is a chronic inflammatory condition linked to several health issues, including cardiovascular diseases, liver diseases, and diabetes. The HG environment associated with diabetic periodontitis leads to more severe outcomes compared to chronic periodontitis. Recent study has shown that pyroptosis plays a significant role in the pathogenesis of periodontitis, particularly through GSDME-mediated macrophage pyroptosis (21). O-GlcNAcylation mediated by OGT in macrophages was significantly increased following treatment with HG and LPS, while OGT knockdown led to a reduction in both O-GlcNAcylation and protein levels of GSDME (22). Furthermore, it was demonstrated that GSDME was significantly upregulated in patients with periodontitis and in macrophages treated with HG and LPS. Additionally, it was found that GSDME knockdown increased cell viability and inhibited the release of inflammatory factors, as well as the protein levels associated with pyroptosis, effectively suppressing macrophage pyroptosis stimulated by HG treatment (22). O-GlcNAcylation mediated by OGT in macrophages was significantly increased following treatment with HG and LPS, while OGT knockdown resulted in reduced O-GlcNAcylation and protein levels of GSDME. Notably, the O-GlcNAc modification of GSDME is exclusively mediated by OGT, occurring on the S339 residue (22). These findings suggest that HG may accelerate the progression of periodontitis by upregulating the expression of GSDME O-GlcNAcylation, thereby promoting macrophage pyroptosis. Another study indicated that GSDME-mediated macrophage pyroptosis is implicated in periodontitis (21). *In vitro* studies demonstrated that *P. gingivalis* LPS induces pyroptosis in RAW264.7 cells through the caspase-3/GSDME pathway (21). However, it remains uncertain whether O-GlcNAcylation affects the interaction between GSDME and caspase-3 in the context of HG periodontitis. Nevertheless, OGT inhibitors could be utilized as a potential strategy to reduce GSDME expression, thereby slowing the progression of diabetic periodontitis.

GSDME can also be indirectly modified by O-GlcNAcylation to regulate cell pyroptosis. Mannose metabolism in the hexosamine biosynthetic pathway increases levels of the metabolite GlcNAc-6P, which binds to AMP-activated protein kinase (AMPK), facilitating its phosphorylation by LKB1. AMPK, a heterotrimeric protein complex, serves as a critical sensor in cellular energy metabolism. Upon activation, AMPK phosphorylates GSDME at Thr6, leading to the inhibition of caspase-3-induced GSDME cleavage, thereby repressing pyroptosis (23). Overall, HG may induce elevated levels of O-GlcNAcylation of GSDME, significantly accelerating the pathogenesis of diabetes-related periodontitis by promoting macrophage pyroptosis.

### 2.3 NLRP3

**Figure 2:**
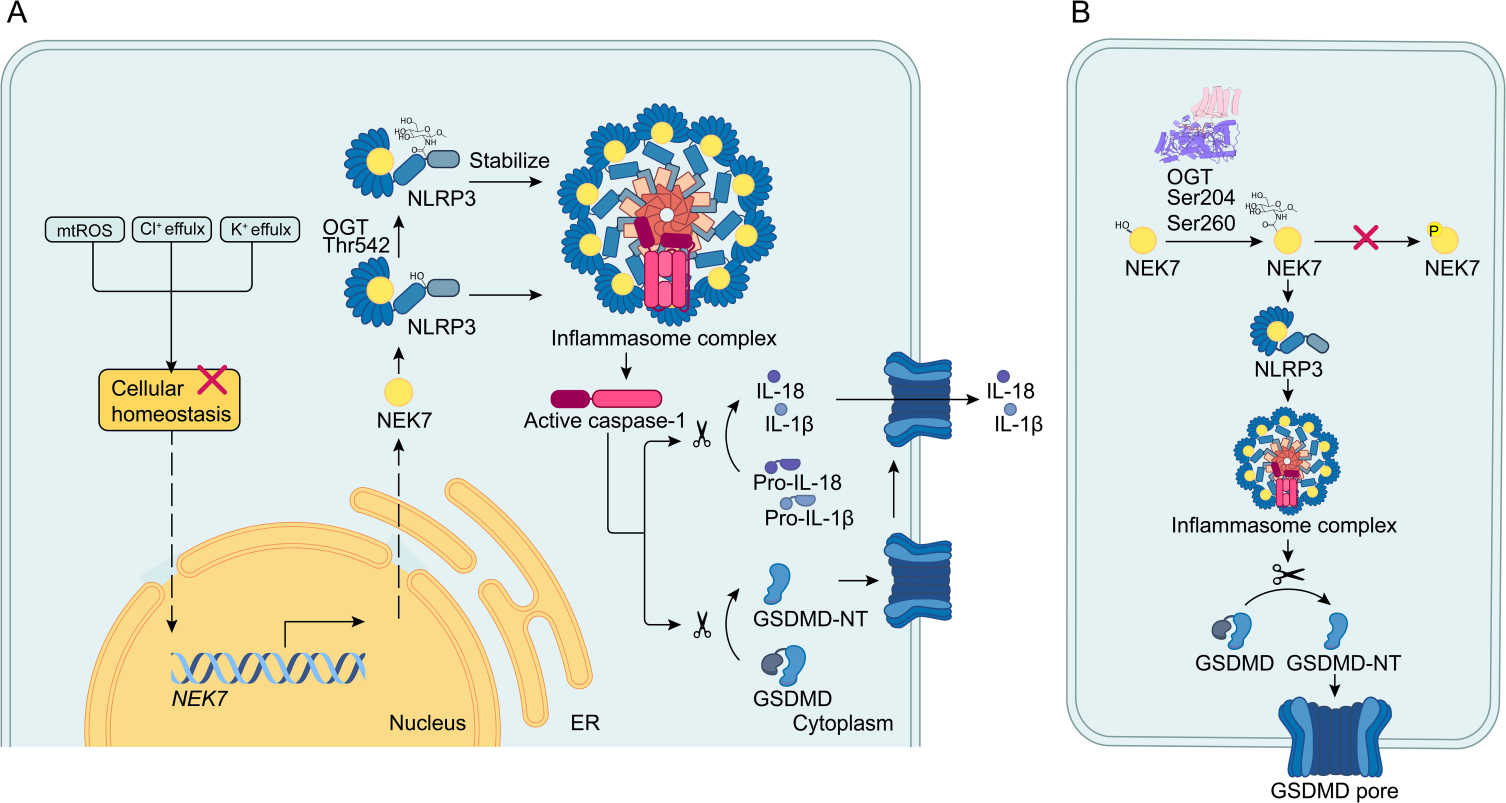
O-GlcNAcylation of the NEK7/NLRP3 axis. **(A)** When cellular homeostasis is disrupted, upstream signals activate the NEK7/NLRP3 inflammasome pathway by increasing NEK7 transcription and enhancing the interaction between NEK7 and NLRP3. The NLRP3 inflammasome then activates caspase-1, which cleaves pro-IL-1β/18 and GSDMD into mature IL-1β/18 and GSDMD-N. GSDMD-N forms pores in the cell membrane, leading to pyroptosis and the release of IL-1β/18. In this process, O-GlcNAcylation of NLRP3 at the Thr542 site stabilizes NLRP3 and promotes pyroptosis. **(B)** Within the NEK7/NLRP3 axis, NEK7 is O-GlcNAcylated at the S204 and S260 sites. O-GlcNAcylation by OGT impedes phosphorylation specifically at the S260 site and enhances the interaction between NEK7 and NLRP3, thereby promoting pyroptosis.

The NLRP3 inflammasome, composed of NLRP3, ASC, and procaspase-1, plays a crucial role in the maturation of IL-1β and the activation of pyroptosis through caspase-1 (**Figure 2A**) (4). Recent research has investigated how O-GlcNAcylation of NLRP3 contributes to pyroptosis induced by LPS in primary human gingival fibroblasts (HGFs), aiming to elucidate the pathogenesis of periodontitis (24). This research indicates that caspase-1, activated by the NLRP3 inflammasome, contributes to the development of periodontitis by inducing cell pyroptosis. Furthermore, the NLRP3 inflammasome is expressed at elevated levels in periodontitis, and inhibiting its activation helps slow the progression of the disease. This suggests that the inhibition of NLRP3-mediated pyroptosis may be beneficial for the amelioration of periodontitis (24). This study confirmed that LPS induces pyroptosis in HGFs and that LPS exposure leads to an increase in O-GlcNAc modification. Notably, the use of the O-GlcNAcylation inhibitor ST045849 attenuated this modification, suggesting that LPS promotes pyroptosis in HGFs by inducing O-GlcNAcylation (24).

Given the significance of NLRP3 in pyroptosis, the study demonstrated that LPS treatment resulted in increased expression of both NLRP3 and OGT, along with a reduction in OGA levels. Additionally, the application of RL2 indicated enhanced O-GlcNAcylation of NLRP3 following LPS exposure. Co-IP and immunofluorescence assays revealed that elevated OGT expression leads to increased O-GlcNAcylation of NLRP3 during pyroptosis (24). Bioinformatics analysis identified three potential O-GlcNAcylation sites on NLRP3: Thr542, Ser159, and Ser199. Notably, mutation at Thr542 significantly reduced NLRP3 O-GlcNAcylation, highlighting Thr542 as a critical modification site (**Figure 2A**) (24).

Interestingly, downregulation of OGT expression dramatically enhanced the degradation of NLRP3, while the OGA inhibitor TMG stabilized NLRP3 at the wild-type Thr542 site in HGFs (24). Further experiments explored the interplay between NLRP3 and OGT. CCK-8 assays and flow cytometry revealed that suppressing OGT increased cell viability in LPS-induced HGFs. Moreover, upregulation of NLRP3 counteracted the reduction of cleaved caspase-1 and GSDMD-N caused by OGT depletion. These findings suggest that LPS promotes HGF pyroptosis by enhancing OGT activity, which regulates NLRP3 through O-GlcNAcylation (24). Applying OGT inhibitors to patients with periodontitis could be an effective treatment strategy.

In parallel, another study explored the impact of OGT-mediated O-GlcNAcylation induced by bisphenol A on NLRP3 stability and cell pyroptosis in non-alcoholic fatty liver disease (NAFLD) (25). This research found that OGT directly interacts with NLRP3 and that silencing OGT significantly promoted NLRP3 degradation. These findings suggest that O-GlcNAcylation mediated by OGT stabilizes NLRP3 protein, thereby accelerating cell pyroptosis (25). More importantly, it sheds light on the involvement of NLRP3 in liver diseases. However, the precise mechanism by which OGT stabilizes NLRP3 remains unclear. O-GlcNAcylation of NLRP3 enhances HGF-induced pyroptosis caused by LPS in the context of periodontitis. Additionally, O-GlcNAcylation modifies the stability of GSDME, contributing to the acceleration of cell pyroptosis in the pathogenesis of NAFLD.

### 2.4 NEK7

NEK7, a member of the mammalian NEK family, plays a crucial role in NLRP3 activation through direct interaction with NLRP3 (**Figure 2A**) (6, 26). As a key regulator of inflammation and pyroptosis (23), recent studies have investigated the role of NEK7 O-GlcNAcylation in regulating cell pyroptosis (27). These studies highlights that pyroptosis has been implicated in the progression of osteoarthritis (OA). Current therapies for OA primarily focus on alleviating clinical symptoms, with no effective methods available to prevent the destruction of articular cartilage during the progression of the disease (27). These studies found that knockdown of OGT decreased the O-GlcNAcylation level of NEK7 without affecting its overall protein level, indicating that NEK7 undergoes O-GlcNAc modification mediated by OGT (**Figure 2B**). To identify functional sites of NEK7 modification, researchers used bioinformatics tools to pinpoint potential sites and then created mutants (T199A, S204A, S234A, and S260A). This approach confirmed that S204 and S260 are critical O-GlcNAc sites on NEK7 (**Figure 2B**) (27). Further investigation into the specific mechanism of NEK7 O-GlcNAc modification led to the hypothesis that O-GlcNAcylation might influence NEK7 phosphorylation. By administering an O-GlcNAcylation inhibitor (ST045849), researchers observed a significant increase in NEK7 phosphorylation and a corresponding decrease in its O-GlcNAcylation (**Figure 2B**). However, whether ST045849 can inhibit OGT to affect the progression of OA remains unclear. Similarly, silencing OGT inhibited the interaction between NEK7 and NLRP3. NEK7 is known to interact with NLRP3 to induce inflammation, and phosphorylation of NEK7 alters this interaction. To determine which sites are specifically involved in phosphorylation changes due to O-GlcNAcylation, researchers constructed S204 and S260 mutants. They found that only O-GlcNAcylation at the S260 site affects NEK7 phosphorylation (27). The application of OGT inhibitors to reduce O-GlcNAcylation of NEK7 may improve the prognosis of OA. In relation to the competition between O-GlcNAcylation and phosphorylation, previous studies have proposed a ‘Ying-Yang model’ to illustrate the interplay between these two post-translational modifications (28). This model demonstrates how O-GlcNAcylation can compete with phosphorylation for the same site or adjacent positions, potentially through steric hindrance. In conclusion, O-GlcNAcylation of NEK7 at the S260 site inhibits its phosphorylation, thereby enhancing the interaction between NEK7 and NLRP3 and promoting the activation of pyroptosis.

### 2.5 NF−κB

The NF-κB family of transcription factors are key mediators of pro-inflammatory gene expression and the inflammatory response (30). Typically, NF-κB exists as a dimer composed of p65 and p50 subunits, which are predominantly localized in the cytoplasm bound to the inhibitor IκB. Upon treatment with TNFα or other activating agents, IκB kinase (IKK) is stimulated, leading to the phosphorylation and subsequent degradation of IκB. This process releases NF-κB, allowing it to translocate to the nucleus and activate target genes (31, 32). NF-κB serves as a key mediator in triggering the priming signal necessary for the activation of the NLRP3 inflammasome (33, 34).

The activation of NF-κB is regulated by post-translational modifications, including phosphorylation and acetylation. Specifically, O-GlcNAc modification of NF-κB plays a critical role in its nuclear localization by disrupting its interaction with IKK (35, 36). Notably, NF-κB O-GlcNAcylation has been implicated in pro-inflammatory roles, influencing inflammatory responses (37). LPS triggers the OGT-dependent O-GlcNAcylation of NF-κB, leading to vascular endothelial inflammatory responses (38). Particularly in rat vascular smooth muscle cells, O-GlcNAcylation of NF-κB p65 at T352 inhibits the interaction between NF-κB p65 and IκB, thereby enhancing NF-κB translocation to the nucleus and increasing VCAM-1 transcription under hyperglycemic conditions (36). Another study shows that OGT-mediated O-GlcNAcylation of NF-κB p65 and IKKα promotes NF-κB signaling activation, TNF-α secretion, and nitric oxide (NO) production in AR42J rat pancreatic acinar cells, potentially exacerbating pancreatitis (39). O-GlcNAcylation of NF-κB also plays a critical role in modulating anti-inflammatory responses during inflammation. Administration of GlcN and PUGNAc has been shown to protect against TNF-α-induced inflammatory stress by enhancing O-GlcNAcylation at the S536 residue of p65 and reducing TNF-α-induced phosphorylation of NF-κB p65. This action effectively suppresses the activation of the NF-κB signaling pathway in rat aortic smooth muscle cells (40).

Supporting evidence has shown that increased O-GlcNAcylation is associated with heightened expression of the NLRP3 inflammasome via the NF-κB signaling pathway in oral lichen planus (OLP) (41). This implies that O-GlcNAcylation of NF-κB is also involved in the NLRP3 signaling pathway. However, the precise mechanisms linking the O-GlcNAcylation of NF-κB and NLRP3 activation remain to be elucidated.

## 3 Conclusions and prospects

Recent research has increasingly highlighted the importance of pyroptosis, linking it to a range of diseases, including cardiovascular disorders, neurological diseases, liver diseases, and tumors. Nevertheless, creating targeted therapies for the key proteins involved in pyroptosis signaling pathways poses significant challenges. One promising area of focus is post-translational modifications, such as ubiquitination (42), phosphorylation (43), palmitoyaltion (44), and O-GlcNAcylation, that affect key regulators of pyroptosis by modifying their interactions, stability, and associated processes.

In our review, we examined the role of O-GlcNAcylation within NLRP3-NEK7 pathway, an essential signaling network governing pyroptosis. We also explored how O-GlcNAcylation interacts with GSDMD and GSDME, two primary executors of pyroptosis, underscoring its potential as a target for manipulating this form of cell death. Despite these insights, several hurdles must be overcome to achieve successful drug development in this area. The impact of O-GlcNAc on these signaling pathways can vary. For instance, O-GlcNAcylation of GSDMD may reduce pyroptosis by hindering its interaction with caspase-4, leading to decreased levels of the active fragment GSDMD-N (17). Thus, effectively modulating O-GlcNAcylation in key regulators such as GSDMD, GSDME, NLRP3, and NEK7 remains a significant challenge for developing therapies targeting pyroptosis. Additionally, pyroptosis plays dual roles in therapeutic responses: controlled pyroptosis aids the body’s defense against infections, whereas excessive pyroptosis can trigger uncontrolled inflammatory responses, potentially leading to inflammatory diseases (45). Consequently, modulating pyroptosis is a critical focus for developing new therapeutic drugs. In the context of O-GlcNAc modification, UDP-GlcNAc serves as the final product of the hexosamine biosynthetic pathway (HBP), which is regulated by several metabolic pathways (46). O-GlcNAcylation is primarily recognized for its role in regulating cellular signaling, transcription, and translation in response to nutrients and stress (47). Proper regulation of O-GlcNAcylation is significant for cellular metabolism. This review aims to summarize recent significant findings on the role of O-GlcNAcylation in regulating pyroptosis, providing new insights into the underlying mechanisms and immune functions associated with O-GlcNAcylation, which are essential for creating effective pyroptosis-targeted therapies (**Table 1**).

**Table 1:** The role of O-GlcNAcylation in various molecules involved in pyroptosis.

Substrate of O- GlcNAcylation	Modification sites	Effects	Diseases	Further research recommendations	Reference
GSDMD	Ser-338	O-GlcNAc of GSDMD attenuates pyroptosis by disrupting interaction between GSDMD and caspase-4, leading to decreased levels of GSDMD-N.	Sepsis	The specific mechanism by which the Ser-338 site of GSDMD affects the interaction between caspases and GSDMD will be further investigated in future studies.	(17)
GSDME	Ser-339	High levels of O-GlcNAcylation of GSDME lead to the acceleration of cell pyroptosis.	Diabetes periodontitis	The precise mechanism by which O-GlcNAcylation regulates the interaction between GSDME and caspase-3 deserves further exploration.	(22)
NLRP3	Thr542	O-GlcNAcylation of NLRP3 promotes cell pyroptosis. Furthermore, O-GlcNAcylation stabilizes NLRP3 in non-alcoholic fatty liver disease, thus enhancing pyroptosis.	Periodontitis and Non-alcoholic fatty liver disease	The exact mechanism by which OGT-mediated O-GlcNAcylation stabilizes NLRP3 remains unclear.	(24, 25)
NEK7	Ser-260	O-GlcNAcylation of NEK7 blocks phosphorylation at the Ser-260 site, which promotes the interaction between NEK7 and NLRP3, thereby enhancing the activation of pyroptosis.	Osteoarthritis	It is still uncertain whether the O-GlcNAcylation inhibitor ST045849 can inhibit OGT and affect the progression of osteoarthritis.	(27)
NF−κB	Thr-352, Ser-536	NF-κB O-GlcNAcylation acts as a crucial mediator in triggering the priming signal required for the activation of the NLRP3 inflammasome.	Pancreatitis, Endothelial inflammatory, Oral lichen planus	The specific mechanism underlying the O-GlcNAcylation of NF-κB in relation to NLRP3 remains unknown.	(38–41)

In the realm of drug development targeting O-GlcNAcylation, several OGT and OGA inhibitors have already been developed (48–50). Since O-GlcNAcylation is regulated by a single pair of enzymes—OGT and OGA—this enhances the accuracy and feasibility of targeted treatments. However, many challenges remain. For instance, the roles of O-GlcNAcylation in pyroptosis depend on specific signaling pathways and the types of diseases involved, which necessitate personalized treatment approaches. For example, the application of OGA inhibitors during sepsis may help improve hypoperfusion (17, 51), while OGT inhibitors could play an anti-inflammatory role in periodontitis (22, 52, 53). Furthermore, the efficacy of current treatments, such as chemotherapy drugs aimed at inducing GSDME-mediated pyroptosis, has been disappointing. This lack of success may stem from GSDME being under-expressed in many tumor cells while over-expressed in normal cells, resulting in reduced effectiveness and potential harm to healthy tissues (54).

Current research into the specific mechanisms of GSDMD O-GlcNAc modification is notably limited. According to Yu’s research, pretreatment with TMG resulted in a significant decrease in the levels of both GSDMD-N and GSDMD-F in HUVECs transfected with LPS, suggesting that O-GlcNAcylation of GSDMD inhibits its cleavage, thus impeding cell pyroptosis (55). However, when LPS was injected into mice followed by intravenous administration of TMG, the expression levels of GSDMD-N aligned with the aforementioned results, but GSDMD-F levels increased. Both GSDMD-N and GSDMD-F are subunits of GSDMD involved in the pyroptosis pathway (56). These discrepancies might be attributed to differences in the timing and methods of TMG treatment. Additionally, Yu’s study did not rule out the possibility of O-GlcNAc modification on GSDME. Since both GSDMD and GSDME are key proteins in the GSDM family linked to pyroptosis, GSDME, when combined with caspase-3, acts as a switch between pyroptosis and apoptosis. The investigation into whether GSDME undergoes O-GlcNAcylation is therefore crucial (55). This research could reveal whether O-GlcNAcylation of GSDME impacts pyroptosis, thus contributing to a more comprehensive understanding of these processes.

The role of O-GlcNAcylation in various diseases has been highlighted, but its impact on NLRP3 in conditions beyond periodontitis and non-alcoholic fatty liver disease remains unclear. Zhang’s research suggests that O-GlcNAcylation of NLRP3 influences its stabilization, thereby accelerating cell pyroptosis (25). Nonetheless, the precise mechanism by which O-GlcNAcylation stabilizes NLRP3 needs further investigation. Previous research has demonstrated that NLRP3 plays a significant role in various diseases, including inflammatory bowel diseases, kidney disorders, liver diseases, Parkinson’s disease, and Alzheimer’s disease (57–61). Despite these findings, studies specifically addressing the O-GlcNAcylation of NLRP3 have primarily focused on periodontitis and non-alcoholic fatty liver disease. The role of O-GlcNAc modification on NLRP3 in other diseases remains largely unexplored. Zhang’s research has shown that O-GlcNAc modification influences the stabilization of NLRP3, which in turn accelerates cell pyroptosis (25). However, the exact mechanism by which O-GlcNAcylation stabilizes NLRP3 has yet to be fully elucidated. Further experimental studies are necessary to clarify how O-GlcNAc modification impacts NLRP3 stabilization.

O-GlcNAcylation exerts influence over various forms of cell death. Previous studies have shown that O-GlcNAcylation accelerates apoptosis in neuroblastoma N2a cells by increasing the levels of cleaved caspase-3, leading to reduced cell viability (46). Additionally, another investigation indicated that O-GlcNAc modification promotes apoptosis by attenuating the phosphorylation and activation of AKT signaling at Thr308 and Ser473 (62). In the case of necroptosis, OGT-mediated O-GlcNAcylation of the serine-threonine kinase RIPK3 at T467 obstructs both RIPK3-RIPK1 and RIPK3-RIPK3 interactions, thereby inhibiting downstream innate immune responses and necroptosis signaling (20). Regarding the regulation of autophagy, O-GlcNAc modification serves as a dual sensor for nutritional and stress-related signals, modulating autophagy by altering O-GlcNAcylation levels to address homeostatic imbalances caused by nutrient and stress conditions (63). In cardiomyocytes, O-GlcNAcylation modifies ULK1, a critical kinase for initiating autophagic flux, and OGT knockout cardiomyocytes exhibit reduced ULK1 O-GlcNAcylation levels, suggesting that the absence of this modification impairs autophagy in cardiomyocytes (64). In summary, O-GlcNAc modification plays essential roles across various forms of cell death, extending beyond its involvement in pyroptosis.

Understanding the impact of O-GlcNAcylation on pyroptosis is still in its early stages, and many aspects remain to be investigated. The influence of O-GlcNAcylation on different tissues and disease states needs to be further examined. Detailed research into the signaling pathways, regulatory mechanisms, and pathological implications of O-GlcNAcylation is crucial. Such investigations will enhance our understanding and could lead to the development of novel and effective treatments for a range of diseases. In summary, while O-GlcNAcylation is known to affect NLRP3 and other related proteins, significant gaps in knowledge remain regarding its broader implications across various diseases. The challenges in drug development and the need for a deeper understanding of the mechanisms involved highlight the importance of continued research in this field. Addressing these issues is vital for advancing therapeutic strategies and improving disease management.
